# 

**Published:** 1874-02-15

**Authors:** 


### *BOOKS RECEIVED,*

*Through Jansen, McClurg & Co., Chicago.*

A Dictionary of Medical Science, with the Accentuation and Etymology of the Terms, and the French and other Synonyms. By Robley Dunglison, M.D., LL.D. A new edition, enlarged and thoroughly revised by Richard J. Dunglison, M.D. Philadelphia: H. C. Lea.

---

Galvano-Therapeutics; a Revised Reprint of a Report made to the Illinois State Medical Society for 1873. By David Prince, M.D., of Jacksonville, Ill. Philadelphia: Lindsay & Blakiston, 1873.

---

Meigs & Pepper on Diseases of Children. New edition.

---

Bellamy's Guide to Surgical Anatomy.

---

Barnes on Diseases of Women.

